# Efficacy of Lavender Essential Oil in Reducing Stress, Insomnia, and Anxiety in Pregnant Women: A Systematic Review

**DOI:** 10.3390/healthcare12232456

**Published:** 2024-12-05

**Authors:** Eulàlia Vidal-García, Maria Vallhonrat-Bueno, Fabià Pla-Consuegra, Alícia Orta-Ramírez

**Affiliations:** Facultat de Ciències de la Salut Blanquerna, Universitat Ramon Llull, 08025 Barcelona, Spain; eulaliavg@blanquerna.url.edu (E.V.-G.); mariavb3@blanquerna.url.edu (M.V.-B.); fabiapc@blanquerna.url.edu (F.P.-C.)

**Keywords:** lavender, essential oil, pregnancy, insomnia, anxiety, stress, phytotherapy

## Abstract

Background/Objectives: During pregnancy, women can experience stress, anxiety, and insomnia, which affect their health and wellbeing. Since many conventional medications are contraindicated for pregnant women, there is a need to find alternative therapies for alleviating their discomfort. Lavender essential oil (EO) is recognized for its calming and relaxing properties; therefore, our goal was to review current knowledge of lavender EO use to reduce anxiety and stress as well as to improve sleep quality in pregnant women. Methods: We conducted a comprehensive literature search in 11 databases that included clinical trials published between 2000 and 2022. Results: Of the 251 articles found, only 6, comprising a total of 413 participants that used lavender EO during the second or third trimester of pregnancy, met the inclusion/exclusion criteria. One trial measured sleep quality, one measured anxiety, two measured both anxiety and stress, and two measured only stress. All studies reported significant (at least *p* < 0.05) improvement in the respective conditions and no adverse effects. Conclusions: The results obtained suggest that although the use of lavender EO during pregnancy has shown to have certain efficacy, given the small number of participants and lack of strong scientific literature, more studies are needed to provide further evidence on this topic.

## 1. Introduction

Phytotherapy is a branch of pharmacognosy and stands for “the use of plants or herbs to treat disease or to relieve pain” [1]. Both traditional and modern use of phytotherapy have shown that plant-derived medicines can alleviate symptoms and help to promote health, more specifically women’s health and wellbeing, including in pregnant, in labor, and postpartum women [2,3,4].

According to the World Health Organization [5], the use of phytotherapy is popular and the primary source of health care in countries with lower accessibility to conventional medicine but is also popular in developed countries due to cultural influence or as complementary therapy, being used by about 70% of Australians and one third of Americans [6].

These plant drugs are crude preparations of dried plants or any part thereof, such as the leaf, stem, root, flower, or seed [7,8].

The European Pharmacopoeia has established a definition of essential oils (EOs): “Odorous product, usually of complex composition, obtained from botanically defined vegetable matter, either by steam distillation, by dry distillation, or by an appropriate mechanical process without heating. An EO is usually separated from the aqueous phase by a physical process which does not lead to a significant change in its composition” [9,10].

EOs can be applied using different routes of administration, such as topical (undiluted to the skin), oral, bathing, diffusion, olfactory, or inhalation [9,11]. Most of the components of EOs are rapidly metabolized and eliminated from the body, so their rapid metabolism and short half-life suggest that they have a minimal risk of accumulation in the body, whatever the route of administration, provided that the recommended doses are respected [12]. It is worth considering the synergistic effect of all the components that make up the EO, as the combined mixture of all the active ingredients leads to an improvement in the efficacy of the total substance [10,13]. At present, EOs are subjected to European guidelines to protect the health and safety of the public [14].

Lavender has been traditionally recognized for its therapeutic properties in the treatment of pain and infections and as a relaxant and sedative [13]. It has also been described to possess antibacterial, antifungal, antidepressant, antispasmodic, calming, and anxiolytic effects, among other properties as well [15,16,17]. The genus *Lavandula* includes more than 30 species, dozens of subspecies, and hundreds of hybrids and selected cultivars and is usually found in the Mediterranean region and Southern Europe [16].

Lavender EO contains 10 major components or active principles belonging to different biochemical groups, differing in percentage. The therapeutic properties attributed to pharmacological actions are due to two organic molecules (which are present in the highest proportion): linalyl acetate (ranging from 25.0 percent to 47.0 percent) and linalool (ranging from 20.0 percent to 45.0 percent) [18].

A study evaluated the effects of *Lavandula angustifolia* EO on various receptors of the central nervous system, and the results showed its affinity for both the glutamate NMDA-receptor and the serotonin transporter (SERT) but not for the GABA_A_-benzodiazepine receptor. The authors concluded that the anxiolytic and antidepressant properties associated with lavender may be achieved by modulation of the NMDA receptor and inhibition of the SERT [19].

Lavender EO is currently recognized and approved by the European Medicines Agency (EMA) as an herbal medicinal product, and its monograph can be found as part of the European Pharmacopoeia [14,18]. Based on the EMA’s description, lavender EO is collected by steam distillation of the flowering tops of the plant *Lavandula angustifolia Mill* (*Lavandula officinalis* Chaix) [18]. According to the WHO monograph on selected medicinal plants, inhalation of lavender EO for symptomatic treatment of anxiety and restlessness and to induce relaxation is safe and supported by clinical data, but due to its traditional use as a possible emmenagogue, it should not be used orally during pregnancy [18,20].

Pregnant women experience situations that can distress them and hinder their well-being, and some EOs have been found useful during pregnancy for the treatment of clinical conditions such as nausea, anxiety, or pain [1,2,3,4]. Since many conventional medications are contraindicated during pregnancy because of their potential for harmful effects on the baby if they cross the placental barrier, it is important to find alternative treatments, and EOs may provide an opportunity, but there are concerns about their applications, safety, and optimal route of administration. Thus, it is important to review the evidence on the safety and efficacy of EOs in pregnant women because, despite the allegedly limited available data, their use has increased worldwide [21,22].

Since the properties and therapeutic effects of lavender EO have been extensively investigated in both animals [23,24] and humans [25,26,27,28,29,30,31,32,33,34] to prove that its use indeed reduces anxiety, increases calmness and relaxation, and aids sleep, it was proposed to examine its potential application more specifically in pregnant women.

Thus, the overarching goal of this systematic review was to evaluate the evidence supporting the effectiveness and safety of the use of lavender EO for the treatment of stress, insomnia, and anxiety in pregnant women to improve their health and wellbeing.

## 2. Materials and Methods

The PICO format was used, formulated as follows: (a) population: pregnant women; (b) intervention: lavender essential oil from the flower; (c) comparison: with control group; (d) outcome: impact on insomnia, anxiety, and stress.

All articles identified through electronic searches were screened independently by two of the authors (A.O.R. and M.V.B.). Any discrepancies in the selection were discussed or, if necessary, referred to a third review author (E.V.G), and inclusion or exclusion was decided upon consensually. The procedure was carried out manually in each of the databases.

### 2.1. Selection Criteria

In this systematic review, the bibliographic search of the studies was carried out using the PubMed, Scopus, CUIDEN, CIBERINDEX, Cuidatge, Biblioteca virtual de salut, ENFISPO, CINAHL, Web of Science (WOS), EMBASE, and SciELO databases, including those studies that were published between 2000 and 2022, and selecting only clinical trials written in English or Spanish.

### 2.2. Search Strategy

The keywords used to conduct the search were obtained from MeSH descriptors, developed by the National Library of Medicine, and the thesauri of the descriptors used in Health Science (DeCS) were used.

For each of these databases, the search was executed using the following combination of terms: for PubMed (“Lavandula”) OR “lavender oil”) AND “Pregnancy”); for Scopus: ((“Lavandula”) OR “lavender oil”) AND (“Pregnancy”) AND (“Anxiety” OR “Sleep Initiation and Maintenance Disorders” OR “Stress, Physiological” OR “Stress, Psychological”)); for CUIDEN: ([res=lavanda]) OR ([res=lavandula]); for CIBERINDEX: ([res=lavanda]) OR ([res=lavandula]); for Cuidatge: ([res=lavanda]) OR ([res=lavandula]; for Biblioteca virtual de salut: (Lavandula) OR (Lavender) AND (pregnancy); for CINAHL: SU (lavender aromatherapy or lavender essential oil) OR SU (lavender and insomnia) OR SU (lavender and anxiety) OR SU lavender aromatherapy AND SU (pregnancy or pregnant or prenatal or antenatal or perinatal or maternal; for ENFISPO: “lavender OR lavandula OR lavender”; for WOS: (Lavandula) OR (Lavender) AND (pregnancy) OR (pregnancy); for EMBASE: lavandula AND pregnancy; and for SciELO: (lavandula) OR (lavender) AND (pregnancy).

Once the search for clinical trials in the previous databases was completed, we proceeded to evaluate a literature review article and a systematic review and meta-analysis of essential oils in pregnant women that had been discarded during the process of analysis of the different databases, as they may have included references of clinical trials of interest that we may not have found previously.

### 2.3. Inclusion and Exclusion Criteria

The inclusion criteria were as follows: (a) clinical trial experiments done with pregnant women of all ages regardless of the number of pregnancies; (b) studies with pregnant women who did not present with concurrent comorbidities; (c) studies that focused on the use of essential oil of the lavender flower; and (d) studies published in either English or Spanish.

This review excluded those studies done in women who had had a previous abortion and/or had suffered from any type of cancer or any other conditions that could deem the pregnancy risky.

We selected studies based on an initial screening of the titles and abstracts and a second screening of the articles’ full text.

**Figure 1:**
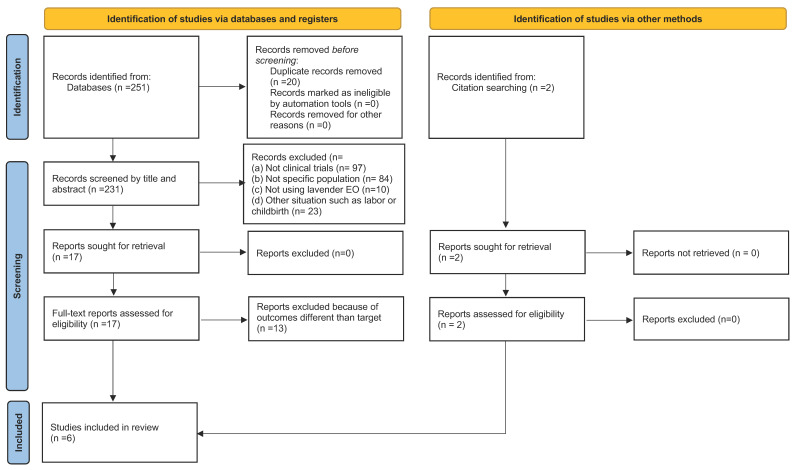
Flow diagram of the systematic review process according to the declarations of the PRISMA protocol. Source: Page MJ, et al. [35].

The identification and the selection of the articles (included and excluded and the reason for their exclusion in the screening and selection phase) are shown in the following flowchart (Figure 1), in compliance with the Preferred Reporting Items for Systematic Reviews and Meta-Analyses (PRISMA) declaration [35].

### 2.4. Quality Evaluation

The quality and risk of bias of the selected studies were assessed by two of the authors (A.O.R and E.V.G) using the same procedures as the study selection.

We followed the guidelines suggested by the Consolidated Standards of Reporting Trials (CONSORT) checklist [36]. Any discrepancies in the evaluations between the two authors were discussed to reach a consensus. For each study, a proportion of fulfilled criteria was assigned. The results of this evaluation are presented in Supplementary Table S1.

### 2.5. Data Extraction

We collected the following data from the selected studies: authors’ name, year and country of publication, type of study, sample size (case/control), age, gestational time, outcome, route of administration, intervention, evaluation, results, and data adjustment (Table 1).

## 3. Results

### 3.1. Results of the Literature Search

The initial search identified 251 references. After removing duplicates, 231 articles were screened by title of abstract, of which 17 were assessed for eligibility reading of the full text. Using previously determined inclusion and exclusion criteria, six articles were selected for this review (Figure 1), from which the data were extracted and summarized using a table (Table 1).

### 3.2. General Characteristics of Reviewed Studies

The analysis of the results considered the type of study conducted, how many women were recruited for the study (n) and in which group they were placed (intervention or placebo), whether they dropped out of the study for any reason, and their chronological and gestational age. In addition, a distinction was made by the outcome studied, the type of treatment the women received, the route of administration used, and what measurement or evaluation instrument was used to assess the effectiveness of the treatment.

All studies were randomized controlled trials [37,39,40,41,42] except one that the authors defined as a “quasi experiment” [38]. Of the six studies selected, one measured sleep quality [37], one measured anxiety [38], two measured both anxiety and stress [39,40], and two measured only stress [41,42].

Sample size ranged from 13 to 141 women for a total of 413 participants, with ages between 18 and 45 years old, and all studies were done during the third trimester of pregnancy, except one trial where the intervention was done in the second trimester [42]. The route of administration of the lavender EO in the studies was either topical [37,39] or by inhalation [38,40,41], except one study where they used a combination of both [42]. When used via topical application, in two studies [37,39], the EO was applied only on the legs using 2 g of cream containing 1.25% lavender, with or without a footbath. In the other study, the EO was applied via a massage of the head, neck, shoulders, arms, waist, back, legs, and feet, using massage oil containing 2% lavender [42]. The control groups in the studies using a topical application received either a placebo cream [37,39] or placebo oil [42]. In the studies using inhalation as the administration route, the case group used either an aroma pendant containing 21 cc of EO [40] or a diffuser containing five drops of EO [41], while the control groups did not. In one of the studies, the patients were exposed to lavender EO (via inhalation) and classical music at the same time, but the study did not have a control group. The measurements were taken before and after the treatment [38].

Five out of the six studies [37,38,39,40,41] recorded the participants’ response to the intervention using different questionnaires: Pittsburgh Sleep Quality Index [37]; Hamilton Rating Scale for Anxiety [38]; Depression, Anxiety, Stress Scale—21 items [39]; State-Trait Anxiety Inventory and visual analog scale [40]; or Profile of Mood States [41]. Only one study used salivary cortisol, measured with an enzyme-linked immunosorbent assay, as an indicator of stress [42]. Two studies were done in Iran [37,39], with one in Indonesia [38], two in Japan [40,41], and one in China [42]. None of the studies reported any adverse effects.

### 3.3. Efficacy of Lavender EO Treatments

Compared to the control group, use of topical lavender EO improved sleep quality in pregnant women (*p* < 0.001) with or without an additional footbath [37]. When combining lavender aromatherapy and classical music, the level of anxiety was significantly lower (*p* = 0.001) after the treatment [38]. Anxiety was also lower after 4 weeks (*p* = 0.002) and 8 weeks (*p* = 0.003) when comparing use of topical cream containing 1.25% lavender with control [39] and after inhalation (*p* < 0.05) using aroma pendants [40].

Use of a topical cream containing 1.25% lavender EO reduced stress after 4 weeks (*p* = 0.006) and 8 weeks (*p* < 0.001) when comparing the case and control groups [39]. When applied topically using a massage oil containing 2% lavender, pregnant women had lower salivary cortisol (indicator of stress) (*p* < 0.001) immediately after the massage and at 36 weeks gestational age (*p* < 0.001) [42]. Inhalation of EOs using aroma pendants or diffusers was effective in reducing stress (*p* < 0.05) in pregnant women [42].

Overall, all studies concurred that administration of lavender EO resulted in a significant (at least *p* < 0.05) reduction of anxiety and stress and improved sleep quality/insomnia of the participants.

## 4. Discussion

In this review, we analyzed the effects of lavender EO on anxiety, stress, and sleep quality of pregnant women based on six articles selected after searching the literature published between 2000 and 2022. This review focused on the use of EO from the flowers of *Lavandula angustifolia* during pregnancy exclusively, not including use during labor or postpartum. Women suffering from cancer and other high-risk conditions as well as women with a previous abortion were also not included in this review.

Based on the results of these studies, lavender EO may be used to alleviate discomfort associated with stress, anxiety, and insomnia, thus improving pregnant women’s health and wellbeing, especially during the second and third trimester of pregnancy. These effects were observed when lavender EO was administered either by inhalation or topical application.

Overall, the results obtained from the review covered a total of 413 pregnant women recruited for the six clinical trials, which may be a small number to reach conclusions beyond the specific conditions of each of the studies. It is worth noting, though, that the results found are consistent with other groups, i.e., there are many other scientific studies in the non-pregnant population that demonstrate the effectiveness of lavender EO in relieving stress, reducing anxiety, and alleviating insomnia [26,27,28,29,30,31,32,33,34].

In terms of limitations, the first and foremost challenge in conducting this review was that although there is plenty of evidence about the positive effects of lavender EO, there is scarce literature on its therapeutic effects during pregnancy, although there have been some studies done during labor and in postpartum. One possible reason of why there is limited research in this area is that pregnant women are a sensitive and at-risk population, thus recruiting them and/or obtaining the approval to conduct trials with this population may be more difficult than with other non-pregnant or non-vulnerable groups, which may have restricted the number of studies that have been conducted in this area.

In two of the studies [40,41], in addition to lavender EO, the authors administered EOs of two other plants (Petitgrain and Bergamot) and pooled together all the results; thus, it was difficult to isolate the effect of lavender EO alone, even though the active principles (linalyl acetate and linalool) were common to all three EOs. Aisyah et al. [38] combined the administration of lavender EO inhalation and classical music; however, the article is missing some information, such as age of the women, comparison with a placebo or control group, type of intervention or concentration/dose, and frequency of application of the lavender EO. Thus, although the authors indicated a statistical significance before and after the intervention with lavender EO and classical music, and they reported a reduction in the anxiety level of the participants, no conclusions can be inferred exclusively for the lavender EO.

This systematic review reflects the interest in a type of treatment beyond conventional pharmacology and the use of safe alternatives to current practice in obstetrics. The prevalence of generalized anxiety disorder (GAD) can be as high as 8.5–10.5% during pregnancy, but pharmacological treatment is only recommended when the benefits outweigh the risks. The use of selective serotonin reuptake inhibitors (SSRIs) and selective serotonin-norepinephrine reuptake inhibitors (SNRIs) may be effective but still controversial given reports of potential side effects on the mother and baby [43]. Benzodiazepines have also been commonly prescribed among pregnant women, but there are serious concerns about the long-term use of these drugs [44]. During this study, we have learned about the importance of conducting studies in pregnant women, as the situation in which they find themselves during pregnancy is one of many physiological and emotional changes, which often cause them to be more stressed or sleep deprived or have periods of increased anxiety; thus, their wellbeing is compromised. On the other hand, it is a population that is very sensitive to certain medications, and thus alternatives to improve their health and wellbeing could be a good option and of considerable help.

The results reported in the six studies selected may not be applicable to all pregnant women, as there may be differences between cultures, genetic discrepancies, or other factors that might make the results vary depending on the location where the clinical trials were conducted. Finally, given the great variation among the interventions (dose, frequency, and type and time of application) and the methods used to measure the outcomes in each of the studies reviewed, caution should be used when considering the results of this review.

Overall, the results obtained show that the studies carried out with lavender EO are statistically very significant and demonstrate that there is a certain efficacy that can be concluded as a positive impact of its use, although it should be noted that much remains to be done in this field. Future studies should be aimed at confirming the safety of lavender EO and establishing guidelines for its use, including, but not limited to, concentration and dosages according to the route of administration, as well as frequency and length of its application during the intervention.

## 5. Conclusions

Through this research, it can be verified that lavender EO may be safe for pregnant women in the specific conditions used in the selected studies, but its use has only been studied from the second trimester of pregnancy onwards, and its safety during the first months of pregnancy is still unknown. From the results obtained from the search, unfortunately, there is not enough information to know whether there may be concentrations of lavender EO that could be detrimental to the health and wellbeing of pregnant women. Topical and olfactory routes of administration are the most used in pregnant women; however, no evidence was found of one route being more effective than the other. In summary, given the lack of scientific literature on the topic of lavender EO use for the treatment of stress, insomnia, and anxiety during pregnancy, we conclude that there is a need for more research to provide safe alternatives to improve the health and wellbeing of pregnant women.

## Figures and Tables

**Table 1:** Detailed summary of studies.

Authors, Year. Country	Study Type Sample Size (n) Case/Control	Age (y) Gestational Time (W)	Outcome	Route of Administration	Intervention	Evaluation	Results	Data Adjustment
Effati-Daryani F et al., 2018. Iran [37]	RCT n:135 (92/43)	18 to 40 y 25 to 28 w	Sleep quality	Topical application on their legs	**Case 1:** 2 g cream (1.25% Lavender) every day and footbath. **Case 2:** 2 g cream (1.25% Lavender) every day. **Control 1:** 2 g placebo cream every day.	PSQI	**Case 1 vs. Control:** −2.9 (−4 to 1.8) (*p* < 0.001) **Case 2 vs. Control:** −2.8 (−4 to −1.6) (*p* < 0.001)	Adjusted for the baseline values and the stratifying factor (parity) used for random allocation
Aisyah et al., 2019. Indonesia [38]	Quasi experiment 50	Third trimester	Anxiety	Inhalation	**Group:** Combination of lavender aromatherapy with classical music	HRS-A	**After vs. before:** −17.82 (*p* = 0.001)	Values not statistically adjusted
Effati-Daryani F et al., 2015. Iran [39]	RCT n:141 (94/47)	18–40 y 25–28 w	Anxiety and stress	Topical application on their legs	**Case 1:** 2 g cream (1.25% Lavender) every day and footbath. **Case 2:** 2 g cream (1.25% Lavender) every day. **Control 1:** 2 g placebo cream every day.	DASS-21	**Anxiety:** **Case 2 vs. Control (4th w):** −2.33 (−3.92, −0.75)) (*p* = 0.002) **Case 2 vs. Case 1 (8th w):** −1.37 (−2.60, −0.15) (*p* = 0.003) **Case 2 vs. Control (8th w):** −1.66 (−2.87, −0.45) (*p* = 0.003) **Stress:** **Case 2 vs. Case 1 (4th w):** −2.30 (−4.13, −0.48) (*p* = 0.006) **Case 2 vs. Case 1 (8th w):** −3.12 (−4.70, −1.53) (*p* < 0.001) **Case 2 vs. Control (4th w):** −1.93 (−3.74, −0.12) (*p* = 0.006) **Case 2 vs. Control (8th w):** −2.70 (−4.27, −1.13) (*p* < 0.001)	Adjusted for the baseline values and the stratifying factor (parity) used for random allocation
Igarashi T and Fujita, 2010. Japan [40]	RCT n:16 (9/7)	29.3 y (case)/27.3 y (control) 26 w	Anxiety and Stress	Inhalation	**Case:** Inhalation of either Lavender, Petitgrain or Bergamot **Control:** No inhalation	STAI VAS	**Trait anxiety (STAI):** Case vs. Control (32nd and 36th w) *p* < 0.05 **Able to relax (VAS):** Case vs. Control (32nd w) *p* < 0.05 Case (32nd vs. 28th) *p* < 0.05 Case (28th vs. 36th) *p* < 0.05	Values not statistically adjusted
Igarashi T, 2013. Japan [41]	RCT n:13 (7/6)	29.3 y (case)/ 27.3 y (control) 28 w	Stress	Inhalation	**Case:** Inhalation of either Lavender, Petitgrain or Bergamot **Control:** No inhalation	POMS	**Case vs. Control:** **T-A** (T = −10.5, *p* < 0.05) **A-H** (T = −10.5, *p* < 0.05)	Values not statistically adjusted
Chen P et al., 2017. Taiwan [42]	RCT n:58 (28/30)	20–45 y (33.31 ± 4.0) 16–26 w	Stress	Topical application via massage of head, neck, shoulders, arms, waist, back, legs, and feet	**Case:** Oil (2% Lavender) **Control:** Placebo oil	Cortisol levels in saliva (ELISA)	**Case vs. Control:** 36 w vs. 16 w post intervention (*p* < 0.001)	Values not statistically adjusted

**RCT**, Randomized control trial; **PSQI**, Pittsburgh Sleep Quality Index; **HRS-A**, Hamilton Rating Scale for Anxiety; **DASS-21**, Depression, Anxiety, Stress Scale—21 items; **STAI**, State-Trait Anxiety Inventory; **VAS**, Visual analog scale; **POMS**, Profile of Mood States; **T-A**, Tension-Anxiety scale; **A-H**, Anger-Hostility scale; **ELISA**, Enzyme-linked immunosorbent assay.

## Data Availability

The data that support the findings of this study are available from the corresponding author upon reasonable request.

## Supplementary Materials

The following supporting information can be downloaded at https://www.mdpi.com/article/10.3390/healthcare12232456/s1. Table S1: Risk of bias judgement according to the Consolidated Standards of Reporting Trials (CONSORT) checklist [36].
