# Supplementary material for: Efficacy of Lavender Essential Oil in Reducing Stress, Insomnia, and Anxiety in Pregnant Women: A Systematic Review
Source: Healthcare (Basel). 2024 Dec 5;12(23):2456. doi: 10.3390/healthcare12232456 (PMC11641599; doi:10.3390/healthcare12232456)
Supplement: Supplementary file 1 [file healthcare-12-02456-s001.zip › healthcare-3272697-supplementary.pdf]

Supplementary Table S1. Risk of bias judgment (high, low, unclear) according to the Consolidated Standards of Reporting Trials (CONSORT) checklist [36].

**Risk of bias domains**		**Effati-Daryani et al.** [37]	**Aisyah et al.** [38]	**Effati-Daryani et al.** [39]	**Igarashi and Fujita.** [40]	**Igarashi** [41]	**Chen et al.** [42]
1a	Title	Low	High	Low	Low	Low	Low
1b	Abstract	Low	High	Low	Low	Low	Low
2a	Background	Low	Low	Low	Low	Low	Low
2b	Objectives	Low	Low	Low	Low	Low	Low
3a	Trial Design	Low	High	Low	High	Low	Low
3b		Low	High	Low	High	High	Low
4a	Participants	Low	Low	Low	Low	Low	Low
4b		Low	Low	Low	Low	Low	Low
5	Interventions	Low	High	Low	Low	Low	Low
6a	Outcomes	Low	Low	Low	Low	Low	Low
6b		High	High	High	High	High	High
7a	Sample Size	Low	High	Low	High	High	Low
7b		High	High	High	High	High	High
8a	Randomization: Sequence Generation	Low	Unclear	Low	High	High	Low
8b		Low	Unclear	Low	High	High	High
9	Randomization: Allocation concealment mechanism	Low	Low	Low	High	High	High
10	Randomization: Implementation	Low	High	Low	High	High	Low
11a	Binding	Low	High	Low	High	High	Low
11b		High	High	High	High	High	Low
12a	Statistical Methods	Low	High	Low	Low	Low	Low
12b		Low	High	Low	Low	Low	Low
13a	Participant flow	Low	Low	Low	Low	Low	Low
13b		Low	Unclear	Low	Low	Low	High
14a	Recruitment	Low	High	Low	High	High	Low
14b		Unclear	Unclear	Unclear	Unclear	Unclear	Unclear
15	Baseline data	Low	High	Low	Low	Low	Low
16	Numbers analyzed	High	Low	Low	High	High	High
17a	Outcomes and estimation	Low	Low	Low	High	High	Low
17b		High	Unclear	Low	Low	High	Low
18	Ancillary analyses	Low	Unclear	Low	High	High	Low
19	Harms	Low	Unclear	Low	High	High	High
20	Limitations	Low	High	Low	Low	Low	Low
21	Generalizability	Low	High	Low	Low	Low	Low
22	Interpretation	Low	Low	Low	Low	Low	Low
23	Registration	Low	High	Low	High	High	Low
24	Protocol	Low	High	Low	High	High	High
25	Funding	Low	High	Low	Low	Low	Low

KEY

High risk of bias (red)

Low risk of bias (green)

Unclear risk of bias (yellow)
